# Characterization of discontinuous ventilation cycles in nymphal *Ixodes scapularis*

**DOI:** 10.1371/journal.pone.0350833

**Published:** 2026-06-24

**Authors:** Samantha Schofield, Jane S. Waters, Y.M. Nuwan D.Y. Bandara, Jannelle Couret

**Affiliations:** 1 Department of Biological Sciences, University of Rhode Island, Kingston, Rhode Island, United States of America; 2 Department of Biology, Providence College, Providence, Rhode Island, United States of America; 3 Department of Chemistry and Biochemistry, Ohio State University, Columbus, Ohio, United States of America; University of Kentucky College of Medicine, UNITED STATES OF AMERICA

## Abstract

Three-host Ixodid ticks, obligate blood-feeding Acari Parasitiformes, spend the majority of their lives off of hosts. They rely on morphological, physiological, and behavioral adaptations to address the compromise between water loss and obtaining oxygen sufficient to meet metabolic needs. Here, we characterize the patterns of ventilation of the blacklegged tick, *Ixodes scapularis*, providing the first measurements for Ixodes, and furthering understanding of the physiology of a medically important vector of tick-borne disease. Measuring CO_2_ output of *I. scapularis* nymphs through a flow-through respirometry system, we confirm discontinuous ventilation in nymphal *I. scapularis*, describe discontinuous ventilation cycles (DVCs), and explore the basis of intraspecific variation rates of CO_2_ emission. Both the frequency of the ventilation cycle and the magnitude of CO_2_ emission bursts varied based on the developmental environment of the nymphs, all F1 laboratory-reared or collected from the wild. We discuss this initial measurement of DVCs in nymphal *I. scapularis* in comparison to other species in Ixodidae as well as the factors which have been shown to modulate the frequency of the ventilation cycles within other species.

## Introduction

Hard ticks are characterized as “gorging and fasting” organisms, being particularly unique in that they can survive longer than any other group of arthropods without access to food or direct access to standing water [1]. They do so via a combination of behavior, active atmospheric water vapor uptake, discontinuous ventilation, and low metabolic rates [2]. The tracheal systems of arthropods, including Ixodid ticks, are efficient for gas exchange [3]. However, water vapor and carbon dioxide are released simultaneously during this process. Therefore, most terrestrial arthropods face a constant compromise between ventilation and desiccation. Indeed, management of this environmental tradeoff is a hypothesized function of discontinuous ventilation in arthropods [4].

Discontinuous ventilation cycles (DVCs) are periodic respiratory rhythms made possible by the regulated opening and closing of the muscular valves to the spiracular openings of the tracheal system. These cycles can generally be characterized as having three phases: a period of low or no gas exchange (the closed phase), a period of rapid spiracular openings (the flutter phase), and a complete opening of the spiracles for a massive CO_2_ release and oxygen uptake (the burst phase). These patterns are not universal. Even among Insecta it is hypothesized that DVCs evolved independently in five different Orders [5]. Few studies have considered ticks and mites, but it is clear that there is no common way in which the Acari respire. For example, Lighton and Fielden [2] found no evidence of DVCs in the giant red velvet mite, *Dinothrombium magnificum,* suggesting this species uses continuous respiration. In contrast, adult *Amblyomma variegatum* displays more typical DVCs, and through exposure to high levels of CO_2_ to induce prolonged opening of the spiracles, it has been shown to relate directly to water loss [6].

Beyond respiration patterns, there are major morphological differences across the sub-class; variation in the presence of spiracles, the number of spiracles pairs present, and their structure and function [7–10]. Within the Order Parasitiformes, respiratory structures are sufficiently diverse to be used as a taxonomic key characteristic for species identification for both nymphs and adult stages [11]. Within the family Ixodidae, gas exchange occurs through a pair of large spiracular plates located on the ventrolateral meridian behind coxae IV. The structure of spiracular plates of Ixodidae has been thoroughly reviewed [12]. In addition to its role in ventilation, there are several hypothesized functions of the spiracular plate, including filtering air to prevent debris from obstructing gas exchange, the reduction of respiratory water loss, the extraction of oxygen from water acting as plastron, and facilitating the survival of ticks under water for prolonged periods [12,13].

Estimating respiratory function of Ixodid ticks expands our knowledge of the diversity of DVCs in a group whose physiology is relatively understudied compared to other major classes in Arthropoda. Here, we estimate the DVC of the nymphal stage of black legged tick, *Ixodes scapularis*. *Ixodes scapularis* is a major vector of tick-borne disease in North America and is known to transmit seven human pathogens [14]. Among these is *Borrelia burgdorferi* sensu stricto, the etiologic agent of Lyme Disease [15,16], which is the most prevalent vector-borne disease in North America. Although all life stages of *I. scapularis* require blood meals for development and reproduction, the primary public health risk is attributed to the nymphal stage blood meal. This is due to a number of factors, including its inconspicuousness due to its small size and the coincident timing of the host-seeking activity in late spring and summer months with outdoor recreational activities in the northeastern and north central United States. Studies have characterized the discontinuous ventilation cycles of *D. andersoni* [17], *A. hebraeum* [18], and other Parasitiformes [2,17]. The intraspecific variation of DVCs in the family Ixodidae, hard ticks, is not well estimated for any species, and *Ixodes scapularis* respiration patterns are not described, with a notable recent exception [19]. Characterization of DVCs of the nymphal stage is both novel and critical towards understanding the most important life-stage due to its role in *Ixodes scapularis*-borne diseases.

## Materials and methods

Wild nymphal *I. scapularis* were collected using the standard methodology of flagging [20] with a ½ m^2^ drag cloth in wooded areas of Kingston, Rhode Island (USA) in June 2022 and kept in humidified (>95% relative humidity (RH)) environmental chambers at 23°C, 14-hour Light: 8-hour Dark lighting schedule. Collected ticks were held at least 10 days in order to ensure they had adequate time to hydrate prior to handling and measurement. Lab-reared ticks were raised from eggs in 3-dram containers that were maintained at 95% RH conditions throughout their lifetime. Larval ticks were fed on *Peromyscus leucopus* and had molted into nymphs a month prior to measurement as approved by URI IACUC Protocol AN08-04-017.

Respirometry measurements were conducted using a flow-through system including a LICOR 7000 Infrared CO_2_/H_2_O gas analyzer in conjunction with Sable Systems UI-2 for analog to digital conversion. The flow rate of dry and CO_2_ -free air from an ultra-zero air compressed gas cylinder was regulated by an Omega mass flow controller (FMA5506; 0–50 SCCM) at 20.4 mL/min. The airstream was hydrated by passing through a section of water-vapor permeable tubing submerged in an airtight water bottle. The respirometry chamber designed to measure individual nymphs was constructed with a minimal volume (approximately 0.1mL) by using short 1/8” nylon luer fittings with a fine stainless-steel mesh to prevent escape (S1 Fig.). Each measurement involved recording a minimum of ten minutes of baseline at the beginning and end of each recording with the same tubing and chamber prior to and after removing the individual tick. CO_2_ concentration and flow rates were recorded at 1 Hz over a period of up to 24 hours for each measurement.

ExpeData v.1.1 [21] was used for data collection and transforming for initial drift correction using beginning and end baselines. Though most ticks were measured for 24 hours, there were several that were measured for a slightly shorter amount of time due to logistics. Therefore, during analysis, all peak datasets were subset to 20 hours for comparison. Raw CO_2_ readings are converted from parts per million (ppm) to $\dot{V}$CO_2_ (where $\dot{V}$CO_2_ represents a rate of mL min^-1^), as outlined in Lighton 2018 using the flow rate (20mL/min) used during observation. The DVCs for each nymph were visualized using the R statistical program [22] with the ggplot2 [23] and photobiology [24] packages. For baseline correction and peak detection, an adaptive baseline-tracking was used (S2 Fig.). Peaks were detected using the ‘msbackadj’ function [25] in MATLAB [26] were overlayed onto the baseline corrected (top) and raw (bottom) datapoints. The package uses a shape-preserving piecewise cubic interpolation for baseline fitting and subtraction. The baseline subtracted dataset is histogrammed and binned through the ‘smoothdata’ function. From that, peaks are found using ‘isoclimax,’ with the peak prominence set to 1/1000 the total data set. These peaks are used to calculate the mean of the peak values for a preliminary baseline mean (mu_prel). Datapoints that are less than mu_prel are isolated. Preliminary baseline standard deviation (std_prel) is then calculated using the standard deviation of the isolated datapoints multiplied by a coefficient (typically between −3 and 10). All datapoints less than mu_prel + std_prel are selected. True baseline mean and standard deviation are then calculate using those datapoints. Peaks are then defined as deviations that are larger than mu_prel+(PDC*std_prel) where PDC is the Pulse Detection Coefficient, which is set between 1–5. Statistical comparisons on the DVCs of 9 lab raised, and 10 wild caught nymphs were run using R statistical program with the stats [22] and effsize [27] packages, using an alpha level (α) of 0.05. A subset of 35 ticks (20 lab raised, 15 wild caught) were weighed (Mettler Toledo ® XPR2 Micro Balance 2.1g x 1µg) prior to the observation period to determine the average weight which was used to create a ratio for allometric comparison of DVCs across genus and species of Ixodidae.

## Results

**Fig 1:**
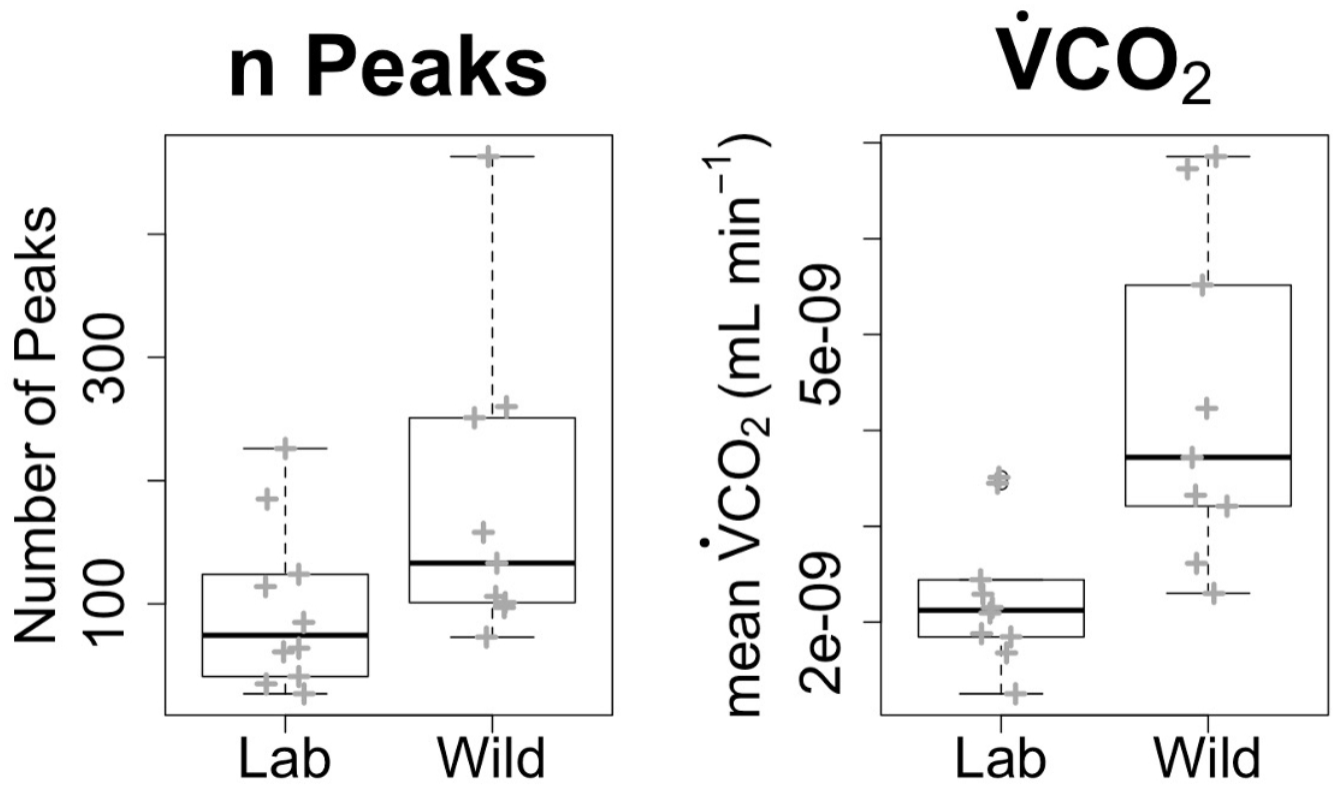
Median number of peaks of lab-reared versus wild-caught nymphal *I. scapularis.* Boxplot of lab-reared (n = 9) and wild-caught (n = 10) ticks comparing the median number of peaks per tick and the average overall $\dot{V}$CO_2_ (over 20-hours) per tick.

**Fig 2:**
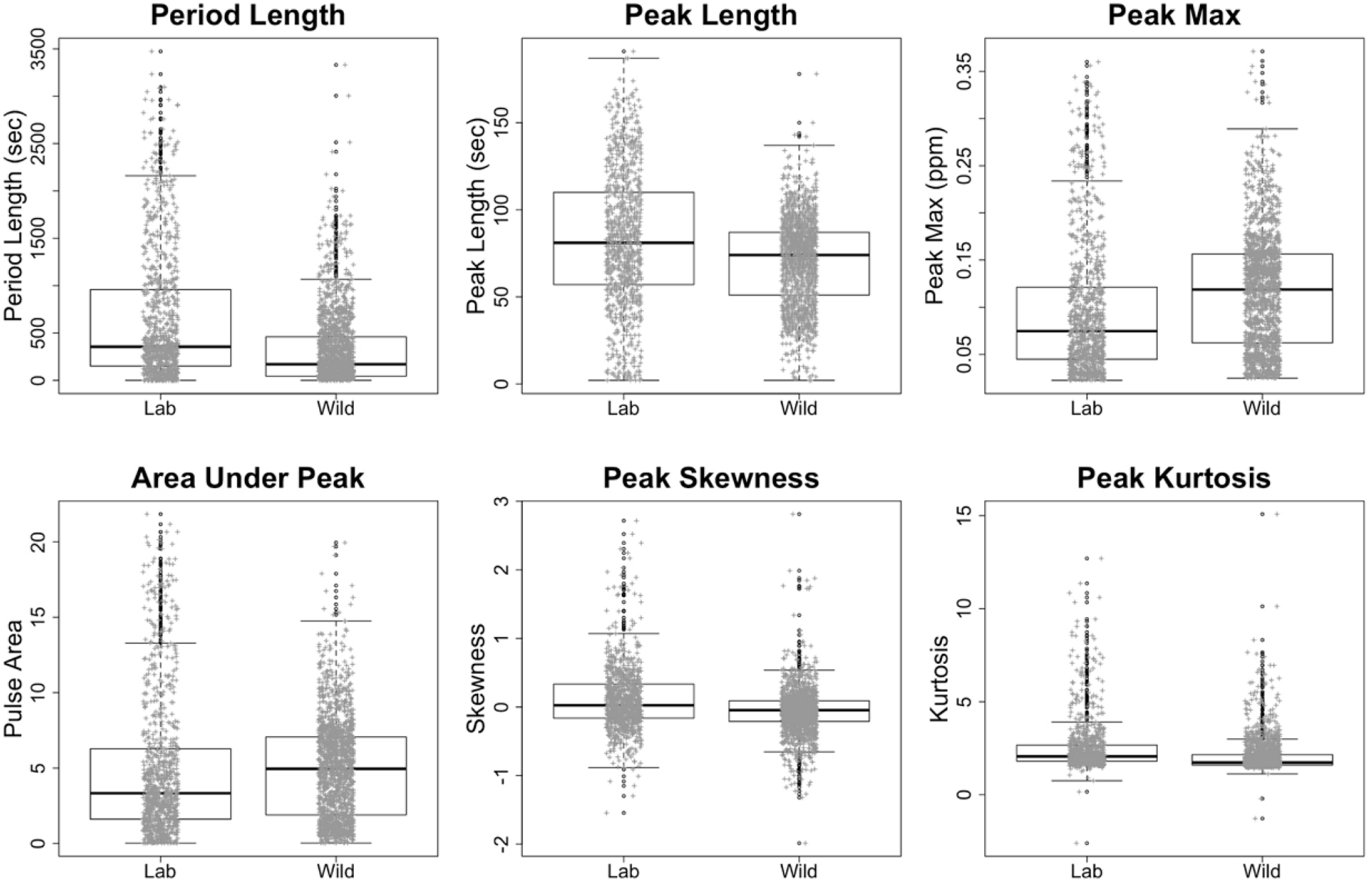
Discontinuous ventilation cycles in lab-reared versus wild-caught nymphal *I. scapularis.* Boxplots comparing features of the DVCs of laboratory reared (n = 9) or wild-caught (n = 10) nymphal *I. scapularis* for 20 hours of measurements of CO_2_ including interburst period length (sec), CO_2_ burst length (sec), max amplitude (ppm), burst skewness, burst kurtosis, and burst area.

Measurements of CO_2_ release of nymphal *I. scapularis* over a 20-hour period in a humidified air stream show regular and periodic 13-minute DVCs, which alternate between the closed phase and the burst phases without strong evidence of a flutter phase. The amplitude, frequency, and shape of the bursts between closed phases are useful for comparison of the ventilation patterns across nymphal cohorts. We found variation in DVCs between wild and lab-reared cohorts of *I. scapularis* nymphs. Ticks collected from the field had more CO_2_ bursts than those reared in the lab (Fig 1, Table 1), with Welch’s t-tests showing statistical clarity between the mean number of CO_2_ bursts in lab-reared vs. wild caught ticks over a 20-hour period (t _(16.933)_ = −2.2984, p = 0.0.03455, d = −1.05). Lab raised ticks had longer periods between peaks (t (_1308.7_) = 13.491, p < 2.2e-16, d = 0.585), as well as CO_2_ peaks that lasted longer periods of time before returning to baseline level (t (_1413.5_) = 9.0288, p < 2.2e-16, d = 0.387). At a flow rate of 20.4 mL/min, the max CO_2_ (ppm) of wild caught ticks was higher than ticks raised in the lab (t (_1815_) = −7.5475, p = 6.979e-14, d = −0.311). The shape of CO_2_ peaks also differed between the two cohorts in both skewness (t (_1513.9_) = 9.7296, p < 2.2e-16, d = 0.413) and kurtosis (t (_1415.4_) = 9.4179, p < 2.2e-16, d = 0.404) (Fig 2, Table 1).

**Table 1:** Comparing discontinuous ventilation cycles of lab-reared versus wild-caught nymphal *I. scapularis.*

	Mean (SE) Lab	Mean (SE) Wild	Median Lab	Median Wild	t (df)	p	d
n Bursts	96 (21.0)	182 (41.5)	75	133	−2.2984 (_16.933_)	0.03455 *	−1.05
$\dot{V}$CO_2_	2.57e −6 (2.296e −10)	1.99e −6 (5.68e −10)	2.13e −6	1.97e −6	−3.7288 (_15.267_)	0.00196 **	−1.74
					**t (df)**	**p**	**d**
Interburst period length (sec)	654 (237.3)	323 (86.1)	354	171	13.491 (_1308.7_)	<2.2e-16 ***	0.585
Burst length (sec)	83 (9.8)	70 (4.4)	81	74	9.0288 (_1413.5_)	<2.2e-16 ***	0.387
Max amplitude (ppm)	0.096 (0.024)	0.12 (0.012)	0.075	0.12	−7.5475 (_1815_)	6.979e-14 ***	−0.311
Burst skewness	0.106 (0.163)	−0.0627 (0.107)	0.0255	−0.0462	9.7296 (_1513.9_)	<2.2e-16 ***	0.413
Burst kurtosis	2.54 (0.051)	2.06 (0.040)	2.06	1.73	9.4179 (_1415.4_)	<2.2e-16 ***	0.404
Burst area	4.98 (1.63)	4.97 (0.61)	3.33	4.96	0.056877 (_1496.4_)	0.9547	ns

*Sig. p < 0.1***.**, p < 0.05 *, p < 0.01 **, p < 0.001 ***, *Not sig*. = ns

T-test comparisons of lab-reared versus wild-caught nymphal *I. scapularis* for the DVC characteristics of interburst period length (sec), CO_2_ burst length (sec), max amplitude (ppm), burst skewness, burst kurtosis, burst area, mean $\dot{V}$CO_2_ (mL/min), and mean number of burst events per tick over 20 hours.

**Fig 3:**
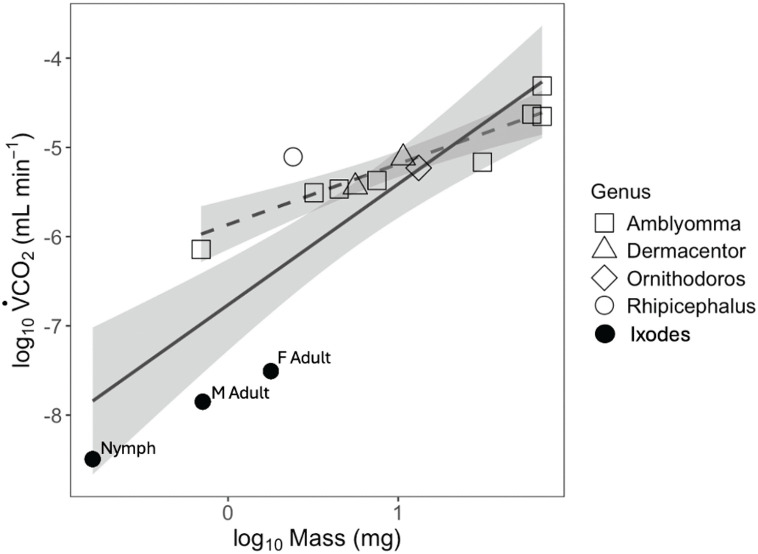
Comparing metabolic rates of multiple genera of ticks. Comparisons of metabolic rates, as log rates of CO_2_ exchange in mL min^-1^ by log mass in mg as reported for multiple tick genera as indicated by character shape (Table 1). Linear regressions, with 95% confidence intervals, were fit with (solid line) and without (dashed line) Ixodes. Data from Lighton et al., 1993 [17]; Lighton and Fielden 1995 [2]; Landulfo et al., 2019 [28]; Marshall et al., 2024 [19]. *I. scapularis* nymphal data collected by author.

Though lab ticks had a lower median of the area under each CO_2_ peak compared to wild caught ticks, the difference between mean of the areas was statistically unclear between cohorts (t (_1496.4_) = 0.056877, p = 0.9547) (Fig 2, Table 1). When comparing the average overall $\dot{V}$CO_2_s’, lab reared ticks had a lower average $\dot{V}$CO_2_ than ticks that were collected from the field (t _(15.267)_ = −3.7288, p = 0.00196) (Fig 1). The relationship between mass and $\dot{V}$CO_2_ was compared with previously reported data (Fig 3, Table 2). The inclusion of *I. scapularis* adults from Marshall et al. 2024 [19] and our reported nymphs resulted in a different linear relationship, increasing the scaling factor from 0.68 to 1.35 (S3 Table).

**Table 2:** Reported masses and metabolic rates of multiple genera of ticks.

Genus	Species	Mass (mg)	$\dot{V}$CO_2_ (mL/min)	Citation
*Amblyomma*	*americanum*	3.2	0.000003.1	Lighton and Fielden 1995
*Amblyomma*	*maculatum*	4.5	3.41667E-06	Lighton and Fielden 1995
*Amblyomma*	*cajennense*	7.5	0.00000425	Lighton and Fielden 1995
*Dermacentor*	*andersoni*	10.7	7.63333E-06	Lighton and Fielden 1995
*Dermacentor*	*variabilis*	5.6	3.61667E-06	Lighton and Fielden 1995
*Ornithodoros*	*moubata*	13.2	5.88333E-06	Lighton and Fielden 1995
*Amblyomma*	*marmoreum*	70.2	2.22333E-05	Lighton and Fielden 1995
*Amblyomma*	*hebraeum*	31.3	6.83333E-06	Lighton and Fielden 1995
*Amblyomma*	*marmoreum*	61	2.35167E-05	Lighton et al., 1993
*Amblyomma*	*marmoreum*	70.2	4.87833E-05	Lighton et al., 1993
*Amblyomma*	*marmoreum*	0.694	7.18333E-07	Lighton et al., 1993
*Rhipicephalus*	*sanguineus*	2.41	7.83333E-06	Landulfo et al., 2019
*Ixodes*	*scapularis*	1.785	3.11167E-08	Marshall et al., 2024
*Ixodes*	*scapularis*	0.7094	1.41383E-08	Marshall et al., 2024
*Ixodes*	*scapularis* (nymph)	0.142	3.21575E-09	

Reported masses and metabolic rates from Lighton et al., 1993 [17]; Lighton and Fielden 1995 [2]; Landulfo et al., 2019 [28]; Marshall et al., 2024 [19]. Nymphal *I. scapularis* data collected by authors.

## Discussion

Here, we characterized the respiration patterns of nymphal *Ixodes scapularis* and explored the basis for intraspecific variation in discontinuous ventilation cycles. The mean interburst period length of nymphal *I. scapularis* was approximately 13 minutes. By comparison, adult *D. andersoni* CO_2_ bursts occur approximately every 121 minutes in dry air, and 139 in humidified air [29]. It is notable that nymphal *I. scapularis* has a length between bursts of less than 10% that of adult *D. andersoni* [29]*,* and approximately 14% of the mean interburst period of adult *A. hebraeum* [18]. Nymphal *I. scapularis* are substantially smaller than adult *D. andersoni* and *A. hebraeum.* Thus, a reasonable explanation for these reduced volumes of CO_2_ expulsion is body size. However, based on allometric comparisons of hard ticks, the DVCs of *I. scapularis* are not comparable between other previously investigated Ixodidae (Fig 3). The estimates from Lighton and Fielden [2] already place tick metabolic rates as lower (12% of the predicted value) than predicted for their body size compared to other terrestrial arthropods, and newly reported adult *I. scapularis* metabolic rates are even lower than those of other reported tick species. When comparing $\dot{V}$CO_2_ and body mass on a log scale, nymphal *I. scapularis* metabolic rates scaled allometrically to those of adults of both sexes recently reported by Marshall et al., 2024 [19] (Fig 3, Table 2). Both nymphal and reported adult *I. scapularis* are outliers in comparison to other reported ticks, which without Ixodes show a scaling factor of 0.68. The inclusion of Ixodes in the regression increases the scaling factor to 1.35 (S3 Table).

Studies have shown that Ixodidae may modulate DVCs, and therefore alter metabolic rate, in response to low relative humidity. The Critical Equilibrium Humidity (CEH) is a species dependent threshold, which estimates place within the range of 75–94% in hard ticks. [30], is the estimated relative atmospheric humidity below which tick survival drops below 50% within 8 hours [31]. The Argasid tick *Ornithodorous concanensis*, showed a 30% increase in metabolic rate when the relative humidity was above the CEH, with the increase in energy expenditure being attributed to active water absorption by relatively dehydrated ticks [32]. A two-fold increase in metabolic rate was observed in dehydrated Ixodid ticks, *D. andersoni*, placed in high humidity environments [29]. Rapid cycling in *D. andersoni,* which is an increase in the rate of cycling, was only observed in dehydrated ticks placed in environments of 95% humidity, suggesting that this stage of ventilation is associated with active water uptake [29]. Indeed, rapid respiratory cycling may be the critical mechanism for atmospheric water uptake to restore water balance [29]. Though such events were observed in some of our observations, they were inconsistent, likely due to the hydrated status of both the nymphs and the air stream (S4 Fig).

Lighton et al. [17] previously reported the absence of a visible flutter phase in adult *A. Marmoreum*. This may be the result of the flutter phase being highly efficient, and undetectable through spiracular CO_2_ loss, or that ticks do not express this phase of DVCs. We similarly could not observe clear increases in CO_2_ during interburst periods and therefore could not categorize a distinct flutter phase. Given that the flutter phase may be untraceable using flow-through respirometry methods, direct observations of spiracles under an electron microscope may shed more light on whether there is spiracular activity in between burst phases.

**Fig 4:**
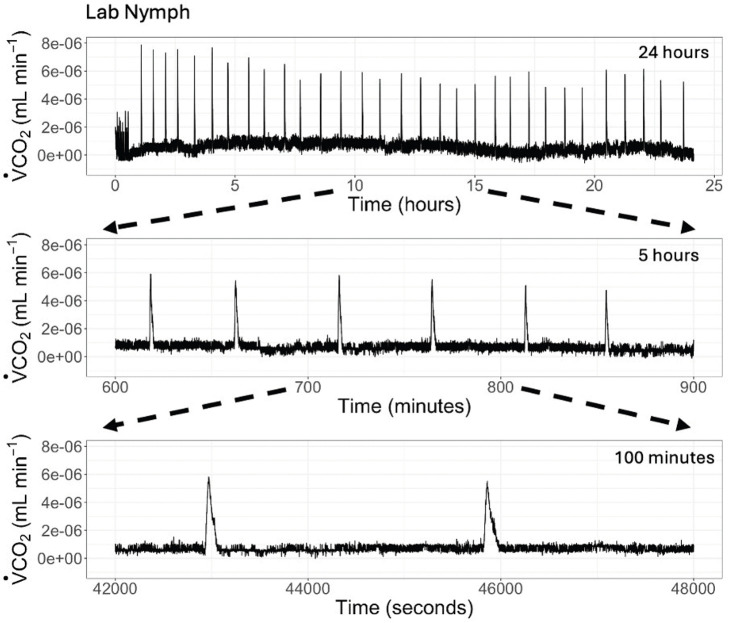
Discontinuous ventilation cycles of a lab-reared nymphal *I. scapularis.* The $\dot{V}$CO_2_ (mL min-1) from a lab-reared nymphal *I. scapularis* tick over 24 hours (top), over 5 hours in minutes (middle), and over 100 minutes (bottom) in seconds. The dotted arrows indicate the time period that is being magnified in each subsequent graph.

**Fig 5:**
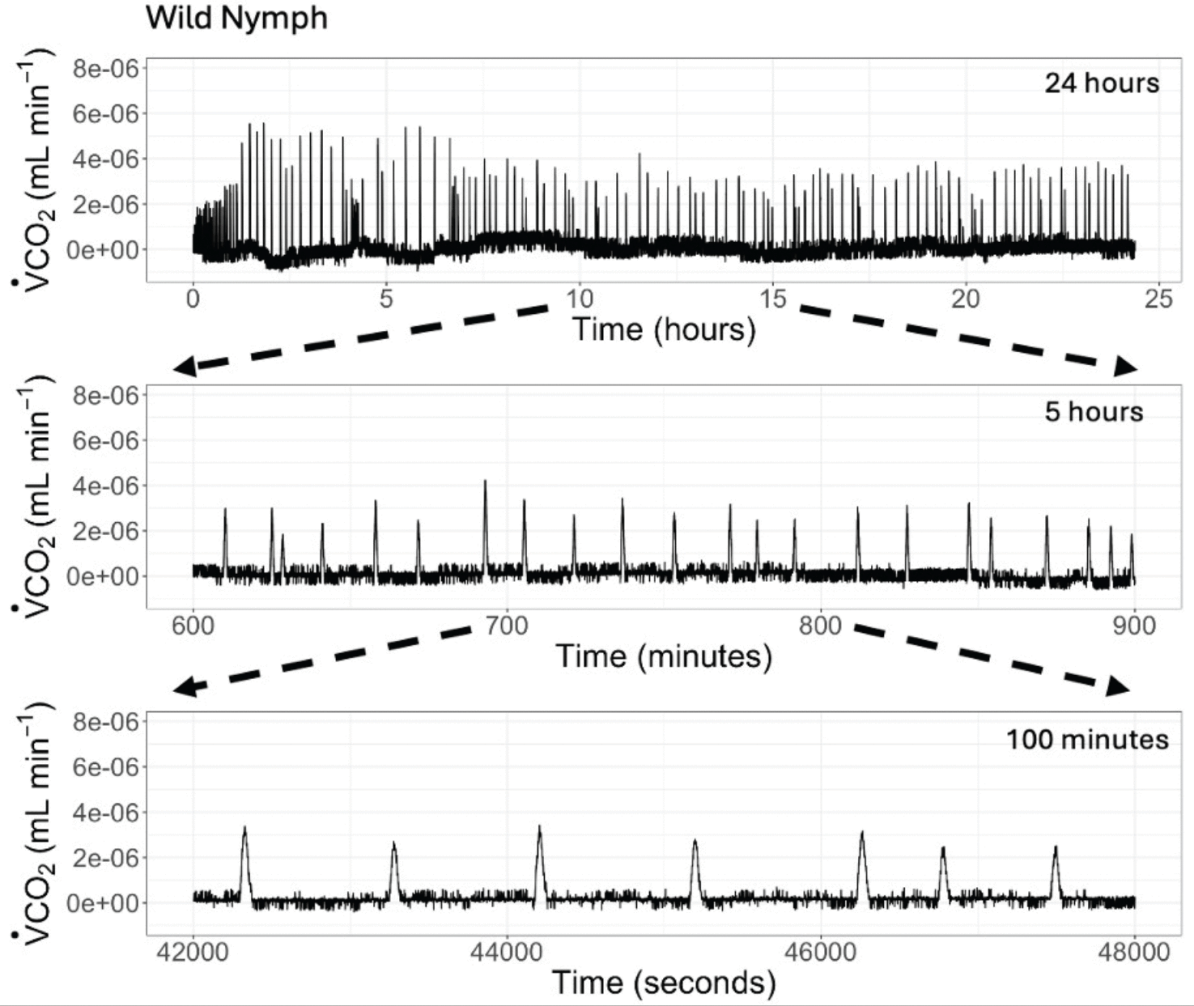
Discontinuous ventilation cycles of a wild-caught nymphal *I. scapularis.* The $\dot{V}$CO_2_ (mL min-1) from a wild-caught nymphal *I. scapularis* tick over 24 hours (top), over 5 hours in minutes (middle), and over 100 minutes (bottom) in seconds. The dotted arrows indicate the time period that is being magnified in each subsequent graph.

**Fig 6:**
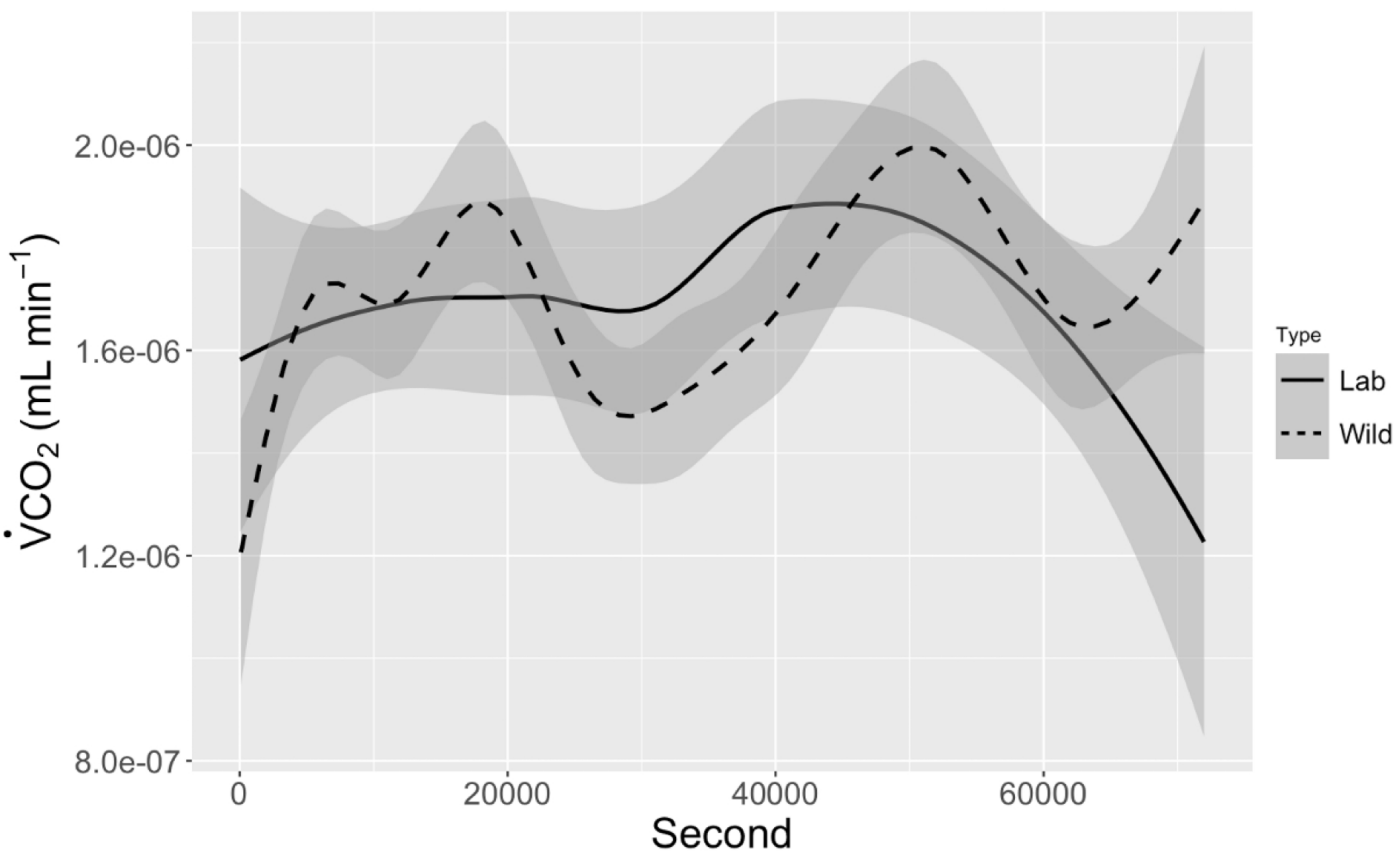
Average $\dot{V}$CO_2_ of lab-reared versus wild-caught nymphal *I. scapularis.* Averages estimated using Locally Estimated Scatterplot Smoothing (LOESS). Means over the observation interval of 20 hours of $\dot{V}$CO_2_ are shown by the solid line for wild (n = 10) and the dashed line for lab-reared (n = 9) *I. scapularis* nymphs, with the 95% confidence indicated by shaded areas. Only CO_2_ levels above baseline were included in the figure.

We explored the basis for intraspecific variation in DVCs by comparing nymphs reared in the lab (Fig 4) to those collected from the field (Fig 5), finding variation on the basis of developmental environment (lab versus field). Lab cohorts differed significantly from field-collected nymphs in DVCs (Fig 2, Table 1). On average, lab-reared ticks only had 53% burst phase events of the ticks collected from the field (Fig 1), suggesting that the rearing environment may influence respiration. Lab reared ticks showed significantly lower $\dot{V}$CO_2_ over a 20-hour period (Fig 1), where the rates also appeared to differ in intensity over the measurement period, with wild caught ticks appearing more varied in intensity, and lab ticks appearing steadier before decreasing towards the end (Fig 6).

Treating each CO_2_ emission as a peak with a normal distribution, kurtosis represents the thickness of the tails. While generally thought of as the frequency of extreme outliers, here it can be interpreted as the length of time that the spiracles remain open relative to a normal distribution. High kurtosis would suggest a longer period of spiracular plate opening and low kurtosis would suggest a more constrained opening and closing. Examining the variation in kurtosis allows us to consider not only the variation in the area under the curve, i.e., the amount of CO_2_, but also the shape of the curve as an indication of the anatomical timing of spiracular opening and closing. By comparing the kurtosis of the two groups (lab versus wild-caught), we are comparing the predictability of the shape of the CO_2_ emissions curve, even in cases when the mean CO_2_ output might be similar. Though both cohorts showed peaks with mean platykurtic distributions, (< 3), CO_2_ peaks of lab reared ticks skewed to the right (positive), where there is less CO_2_ buildup before reaching the peak, unlike wild caught which skewed left (negative), so had a longer build up before reaching the max peak value (Fig 2, Table 1).

Given that both cohorts were exposed to the same high-humidity environment for at least 10 days to facilitate rehydration prior to measurement in a humid airstream, these results suggest the rearing environment may have a cumulative impact on respiration patterns of *I. scapularis* ticks subjected to the same external conditions. The estimation of DVCs in *I. scapularis* nymphs provides a novel measure to understand the known phenotypic impacts of temperature and relative humidity on tick [33–35] and life-history traits [30,36]. Future investigations of *I. scapularis* may incorporate environmental factors of heat and desiccation stress to explore the relationships between abiotic environmental conditions and tick metabolic rates.

## Supporting information

S1 Fig Respirometry chamber setup. The chamber setup used for flow-through respirometry measurements. Chamber size was approximately 0.1mL. The chamber was closed using wire mesh, which allowed for airstream to move through.
(TIFF)

S2 Fig CO_2_ peak detection. Side by side comparison of the raw data of CO2 output, and the baseline corrected plot over 20 hours. The blue line is the baseline, the red line is the threshold for a spike to be considered a peak, and the pink are where peaks were detected.
(TIFF)

S3 TableLinear regressions of metabolic rates across genus of ticks including and excluding I. scapularis.Linear regressions comparing previously reported $\dot{V}$CO_2_ (ml min^-1^) and mass across multiple genus of ticks (Fig 1, Table 2). Regression A (left) does not include *Ixodes scapularis*. Regression B (right) includes adult male and female *I. scapularis* reported by Marshall et al., 2024 [19], and our measurements of nymphal *I. scapularis*.(TIFF)

S4 Fig Observed respiration event in a wild caught nymphal *I. scapularis.* An example of a potential Active Respiration Event, as described in Fielden and Lighton 1996 [29], in a wild caught nymphal *I. scapularis* zoomed in at three different scales.
(TIFF)

S5 Data Microsoft Excel spreadsheet of the complete respiration dataset recorded and analyzed in this work. (XLSX)
