# Supplementary figures and images for: Characterization of discontinuous ventilation cycles in nymphal Ixodes scapularis
Source: PLoS One. 2026 Jun 24;21(6):e0350833. doi: 10.1371/journal.pone.0350833 (PMC13293453; doi:10.1371/journal.pone.0350833)

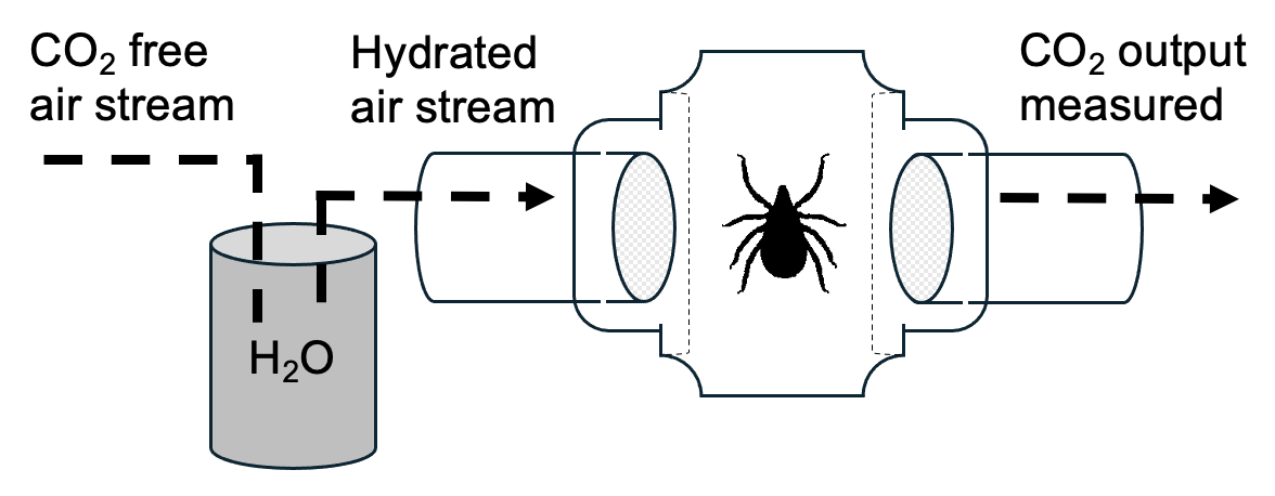

Supplement: S1 Fig — The chamber setup used for flow-through respirometry measurements. Chamber size was approximately 0.1mL. The chamber was closed using wire mesh, which allowed for airstream to move through. (TIFF) [file pone.0350833.s001.tiff]

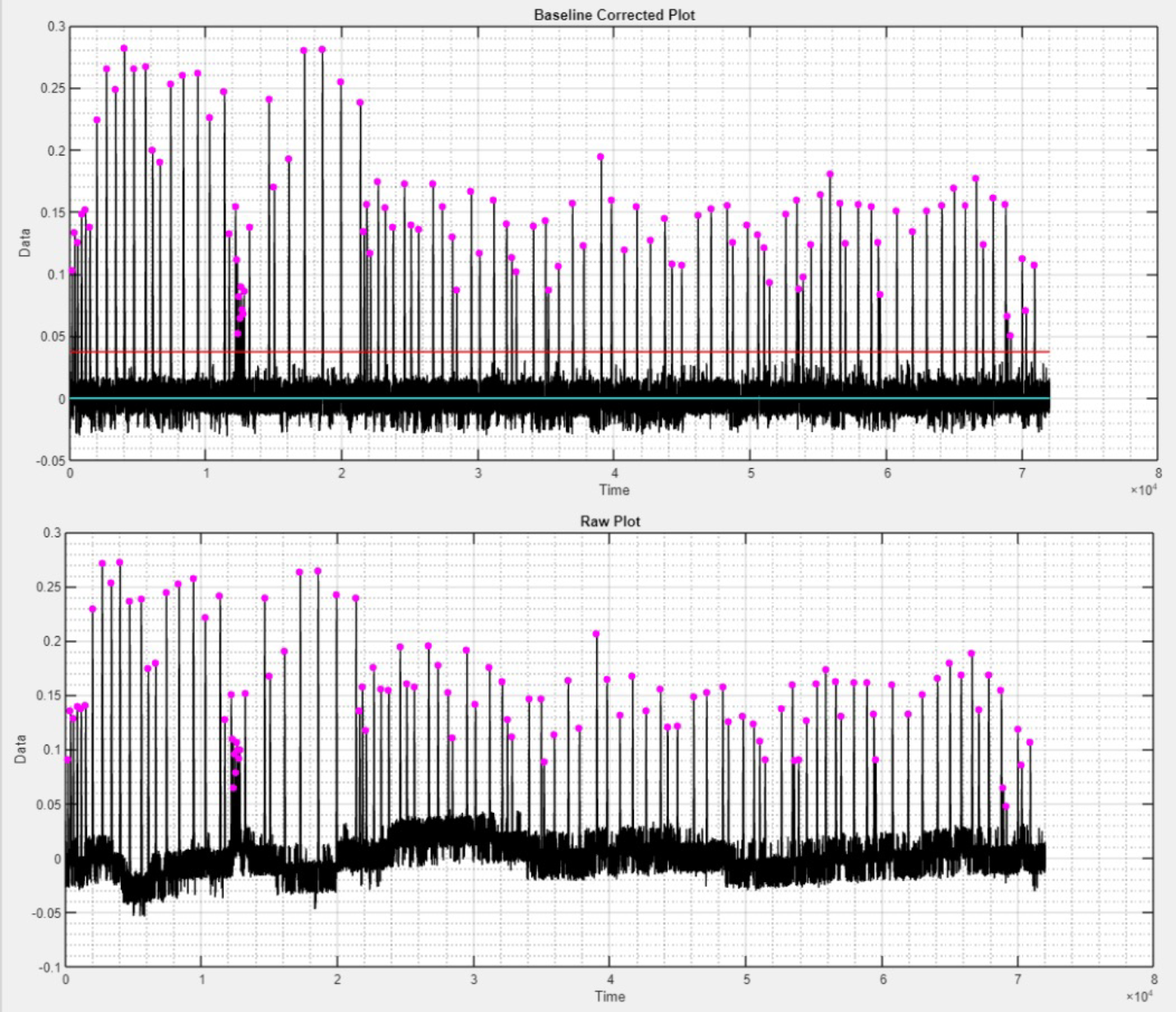

Supplement: S2 Fig — Side by side comparison of the raw data of CO2 output, and the baseline corrected plot over 20 hours. The blue line is the baseline, the red line is the threshold for a spike to be considered a peak, and the pink are where peaks were detected. (TIFF) [file pone.0350833.s002.tiff]

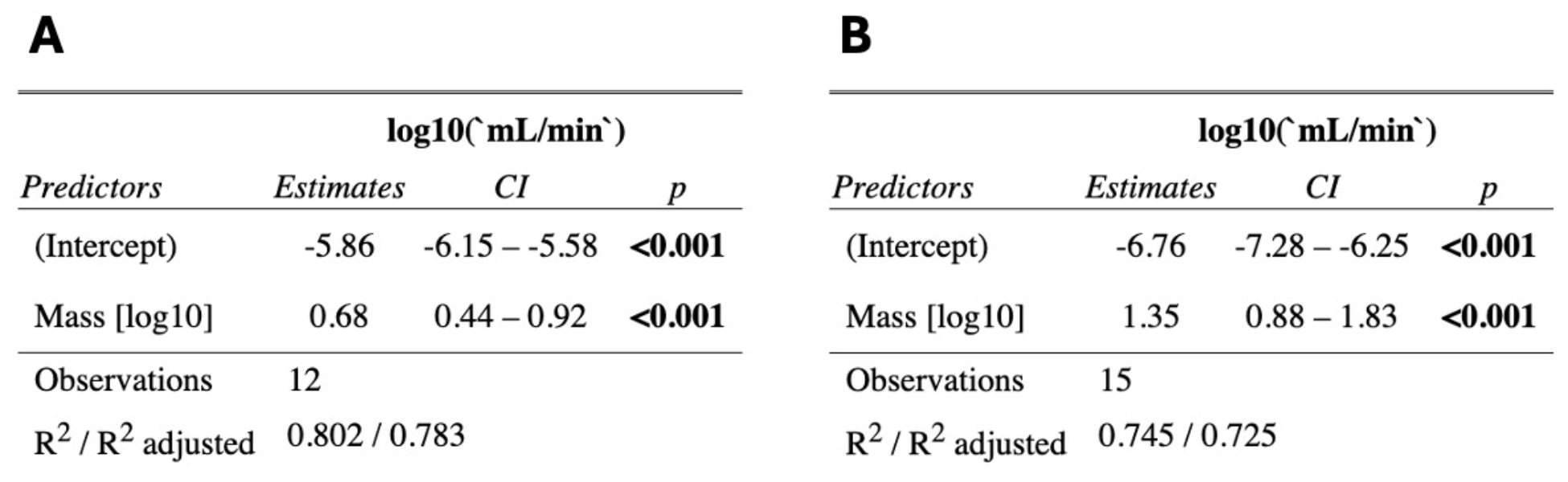

Supplement: S3 Table — Linear regressions comparing previously reported V˙CO2 (ml min-1) and mass across multiple genus of ticks (Fig 1, Table 2). Regression A (left) does not include Ixodes scapularis. Regression B (right) includes adult male and female I. scapularis reported by Marshall et al., 2024 [19], and our measurements of nymphal I. scapularis. (TIFF) [file pone.0350833.s003.tiff]

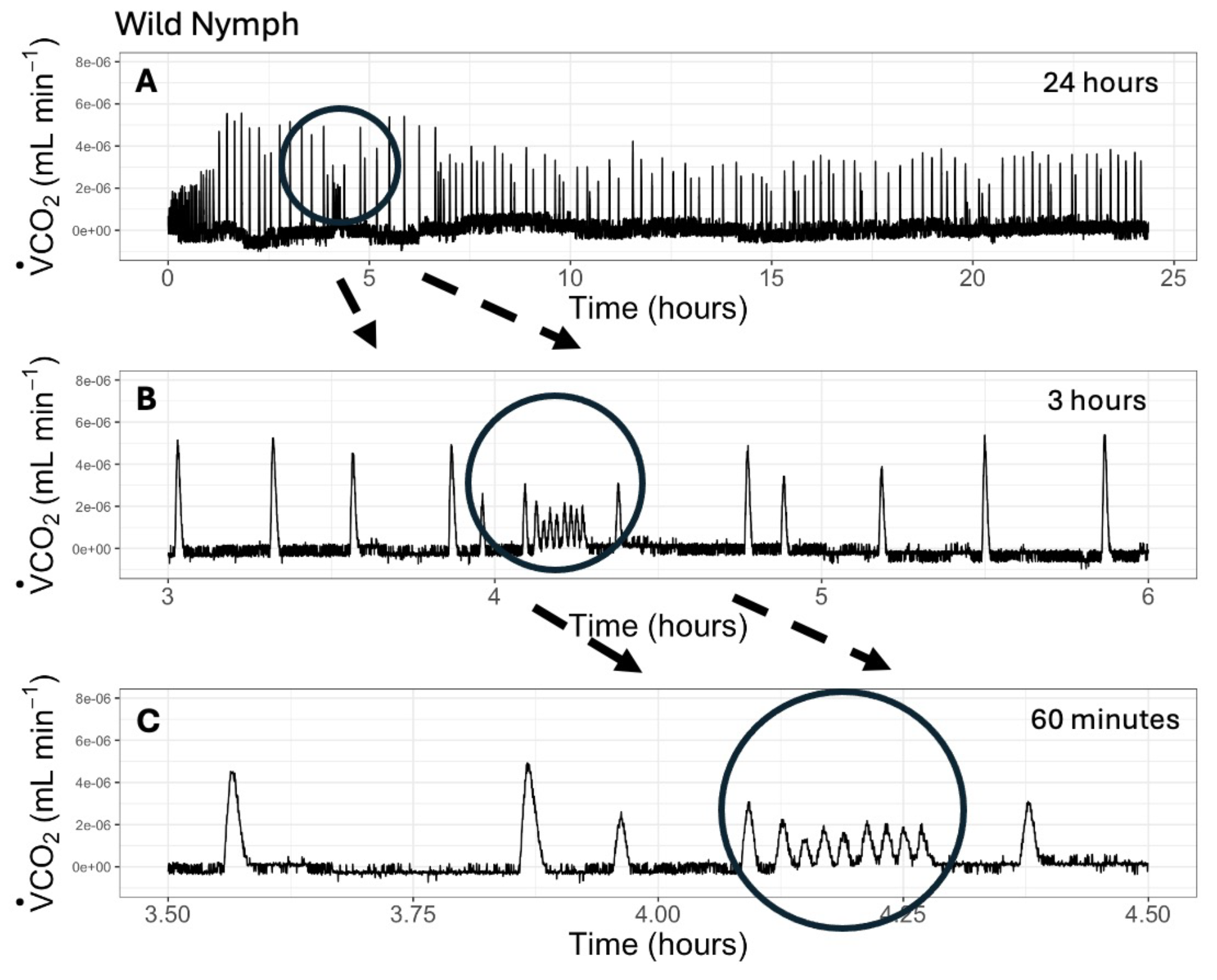

Supplement: S4 Fig — An example of a potential Active Respiration Event, as described in Fielden and Lighton 1996 [29], in a wild caught nymphal I. scapularis zoomed in at three different scales. (TIFF) [file pone.0350833.s004.tiff]
